# Intrathecal Baclofen Infusion Pump for the Treatment of Painful Spastic Hemiplegia: A Case Report

**DOI:** 10.7759/cureus.44503

**Published:** 2023-09-01

**Authors:** Juan J Medina-Pérez, Andrés Vega-Rosas, Rubén A Martínez-Espinosa, Daniel Chávez-González, Silvia G Coubert-Pelayo

**Affiliations:** 1 Pain Management Center, Hospital Ángeles Mocel, Mexico City, MEX; 2 Pain Clinic, Hospital Escandón, Mexico City, MEX; 3 Anesthesiology Service, Hospital Ángeles Mocel, Mexico City, MEX

**Keywords:** interventional pain medicine, intrathecal pump therapy, chronic pain management, stroke complications, spastic hemiplegia

## Abstract

Painful spastic hemiplegia is a common sequel to a stroke in which patients rarely achieve optimal levels of pain control. Herein, we report the case of a 62-year-old woman with painful spasticity secondary to an ischemic stroke of 15 years' evolution who received multiple pharmacological treatments without reaching motor or pain management goals. After an adequate analgesic response to the intrathecal baclofen test, the placement of an electromechanical pump was decided, reaching an effective maintenance dose of 150 μg per day. Despite achieving partial improvement in spasticity, optimal pain remission was achieved.

## Introduction

Spasticity is a common neurological sequelae of stroke that affects 25.3% of patients [1], with direct repercussions on their sensory and motor functionality, significantly reducing quality of life when it is associated with chronic pain. As a motor disorder characterized by a velocity-dependent increase in muscle tone or tonic stretch reflexes associated with hypertonia [2], spasticity is a type of upper motor neuron syndrome that manifests clinically as an increase in tonic stretch reflexes, causing weakness, reduced motor control, pain, spasms, and abnormal posture [3-5]. The treatment of hemiplegia after a stroke is essential, and rehabilitation [6], as well as other interventions [7,8], offer interesting results on the motor skills of the affected muscle groups. However, the painful nature and the sensory alterations that accompany spastic hemiplegia continue to represent a therapeutic challenge that does not always reach goals [9], especially because the neuroplastic changes secondary to the pain syndrome complicate its treatment when they become chronic and are not treated promptly.

Like all neuropathic pain, its treatment with anti-inflammatories is ineffective and prone to side effects. Among the pharmacological interventions to improve pain associated with spastic hemiplegia, the use of focused chemodenervation with type A botulinum toxin has been standardized, demonstrating relief and reduction of spasticity; however, its effectiveness is time-dependent [10,11]. Another innovative and recently used alternative is extracorporeal shock wave therapy, which offers spasticity relief with minimal invasion; however, it presents a lower spasticity reversal rate (17%) compared to baclofen infusion (29%) [12]. Baclofen is a GABA-B receptor agonist that inhibits reflexes in the spinal cord, effectively reducing spasticity. Intrathecal administration has proven to be the safest, as it presents fewer and less intense spasms, especially in patients with pain refractory to other treatments [13]. Although opioids and baclofen are safe and effective in treating chronic neuropathic pain, the response to these treatments has become imprecise over the years. Intrathecal Baclofen Infusion Pump (IBIP) therapy is the most widely accepted management due to the substantial improvement in spasticity in the upper and lower limbs [14,15], due to its high bioavailability, reducing the amount and severity of muscle spasms caused by neurological damage, improving muscle tone homeostasis, and reducing the pain associated with hypertonia. Additionally, it allows adjusting the doses according to the specific needs of the patient with a wide margin of safety due to its reversibility.

## Case presentation

A 62-year-old woman with a history of painful spastic hemiplegia of the left side of the body that presented secondary to an ischemic stroke of 15 years' evolution. No other relevant pathological history. Over time, she was evaluated by multiple healthcare providers, including orthopedics, neurology, neuropsychology, and rehabilitation, without successfully mitigating the pain syndrome. She received multiple treatments focused on reducing stiffness and frequency of spasms, as well as pain control with tramadol, tizanidine, non-steroidal anti-inflammatory drugs, paracetamol, cannabidiol drops, muscle relaxants, calcium, and even the application of botulinum toxin, with which she presented side effects characterized by persistent flaccidity. Additionally, she was medicated with oral baclofen titrated to a maximum dose without obtaining a satisfactory response. Likewise, elbow and hamstring tenotomies have been performed to alleviate spasticity, with partial improvement.

After being classified as having intractable pain, she decided to go to a pain management center for a medical evaluation for interventional algology. A clinical presentation of left hemiplegia was found to be characterized by shoulder adduction and internal rotation with a flexed arch, flexed elbow, pronated forearm, flexed wrist, clenched fist, thumb stuck to palm deformity, excessive hip flexion, adduction of the thighs, extended knee, clubfoot, and hyperextension of the big toe. In a catheterization room with fluoroscopy, a trial of intrathecal infusion of baclofen in a single dose of 100 μg was performed, presenting immediate improvement in spasticity and pain. The test was considered positive, and upon corroborating that she was a candidate for intrathecal infusion treatment with an IBIP, the procedure was scheduled.

**Figure 1:**
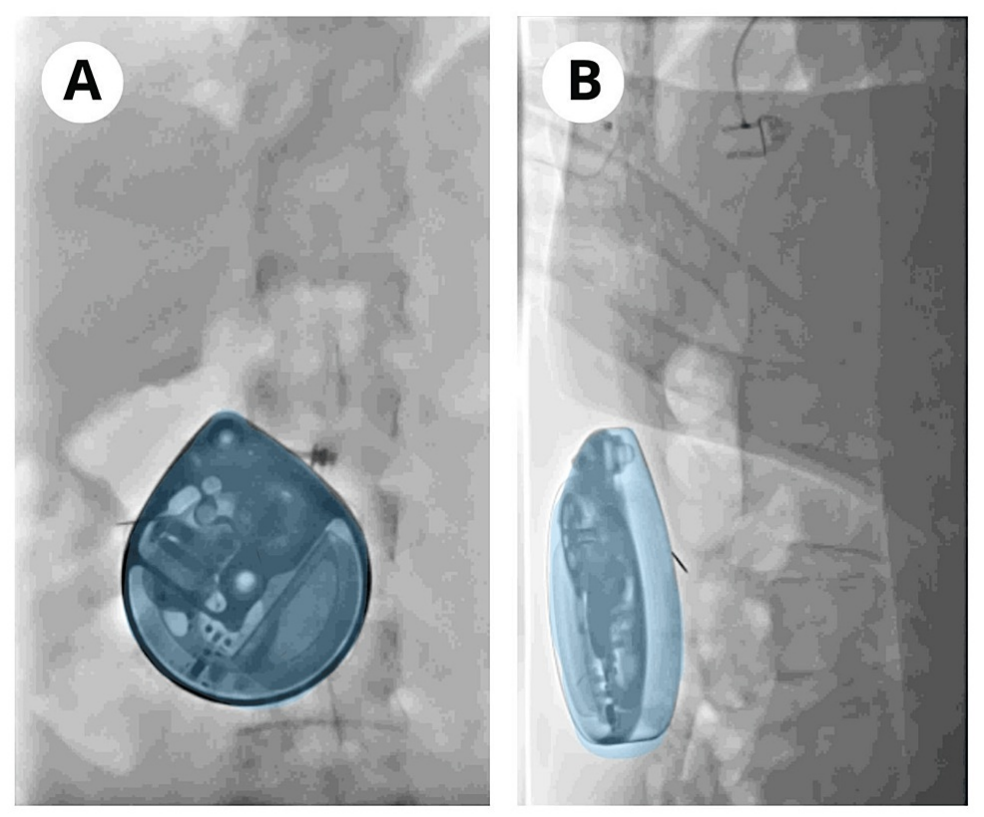
Definitive implantation of the electromechanical pump The device is observed in blue at the right subcostal level in subcutaneous tissue, in its anteroposterior view (A) and in its lateral view (B), through fluoroscopy in real time while it is being placed.

Under general anesthesia and in the prone position, a fluoroscopy-guided approach to the L2-L3 space was performed. Under local anesthesia, a guided puncture with an intralaminar approach was made up to the subarachnoid space, from which cerebrospinal fluid was obtained. The intrathecal catheter was advanced through fluoroscopic vision up to the T10 region, where it was fixed to the aponeurosis. The subcutaneous catheter was tunneled from the lumbar region to the right flank of the abdomen, and the patient was positioned supine, maintaining general anesthesia and endotracheal intubation. Subsequently, with local anesthesia, a right subcostal incision is made, and the aponeurosis is incised to make a pocket for the definitive implantation of the Medtronic SynchroMed™ II pump, consisting of a battery-powered programmable pump with a reservoir, which is filled with 20 ml of baclofen at a concentration of 1000 μg/ml, and is programmed in continuous intrathecal simple mode injection, with an initial bolus of 50 μg for a total dose of 100 μg in 24 hours (Figure 1). The procedure ended without eventualities or complications after a surgical time of 90 minutes and 120 minutes of immediate postoperative recovery to proceed to hospitalization.

An evaluation was carried out the following day, in which the patient already presented a partial improvement of her symptoms to more than 80% by goniometry, Measurement of Motor Function by Palisano, Barthel Index, and Visual Analog Scale score with respect to spasticity and pain. She was discharged from the hospital 72 hours after surgery due to improvement and clinical stability, with a decrease in the frequency of spasms (zero points on the Penn Spasm Frequency Scale) and remission of pain. Intensive physical rehabilitation therapy has been started since the first week of discharge. Subsequently, clinical evaluations were performed every month for three months. A 25% increase in the initial dose of baclofen was required each month, reaching a maintenance dose of 150 μg of baclofen for 24 hours. The only adverse effect observed was a change from painful spasticity to severe hypotonia (−3 points in the classification of Campbell Scale hypotonia). However, the patient's quality of life has improved, as well as the mobility of the left leg, although flaccidity in the arms has persisted, with optimal pain remission.

## Discussion

Although the effectiveness and safety of the use of IBIPs for spasticity are well known, there are few case reports that focus on the improvement of the chronic pain that accompanies this condition [12,15], especially when the evolution of the same is greater than ten years. Spastic hemiplegia is a very common condition in patients who have suffered a stroke and is often accompanied by chronic pain that is rarely optimally treated. Among the most common pain syndromes in this context is shoulder pain, which is reported in up to 40% of patients but may be observed in as many as 90% of post-stroke patients with hemiplegia or those receiving rehabilitation [16]. The highest incidence of pain occurs within the first six months post-stroke, although it may initially occur as early as one week or at up to 16 months [3]. In the present case report, as well as in the vast majority of patients with chronic neuropathic pain associated with an ischemic event, they go through the pilgrimage of multiple physicians and other health providers to seek relief from their pain without achieving the results desired. Pain medicine and interventional algology remain inaccessible areas of medicine in many parts of the world, such as Latin America. In Mexico, although there are no specific statistics for painful hemiplegia, the absolute number of stroke new cases and deaths increased up to 75.3% in the last 29 years [17], so the parallel increase in painful syndromes that do not receive optimal care has surely also been presented.

Spasticity, such as an imbalance in supraspinal inhibitory and excitatory inputs as well as a hyper-excitable state of the stretch reflexes, requires neuromodulatory treatment to effectively manage its symptoms. Among the most commonly used therapeutic strategies are physical therapy, splinting, oral medications, chemical neurolysis, and surgical interventions [6,18], whose effectiveness will be very heterogeneous. As in the present case, different anti-inflammatory and opioid analgesic schemes were used for 15 years, as well as physical rehabilitation, without real improvement in pain. It is worth questioning whether there was really adequate adherence to treatment when these different modalities were tried; however, it can be concluded that none was effective enough for the patient to have maintained a specific treatment until her pain was resolved, preventing it from becoming chronic. It is also important to consider that when pain management goals are not reached, the evolution is accompanied by the aging of the patient, the rising prevalence of comorbidities, and a decrease in functional capacity. Therefore, implementing timely pain management interventions and providing advice for nurses caring for patients who are experiencing this type of pain when it becomes chronic [19] begins to elucidate the need to generate public policies aimed at prevention.

Despite the promise of IBIP-based treatment to improve spasticity, posture, sleep pattern, sweating, and chronic pain, it does not cease to represent a potential risk for adverse effects that must be considered individually in each patient [14]. However, unlike orthopedic musculoskeletal surgery and selective posterior rhizotomy, which are irreversible, the effect of IBIP can be modulated dynamically to achieve the best relief of discomfort with the fewest adverse effects or even to discontinue treatment at any time without further limitation [20]. In the present case, although the patient showed total pain relief and partial spasticity improvement in her left leg, she developed flaccidity in both arms, which were also non-functional prior to the intervention. The only relevant adverse effect presented was severe hypotonia that will become chronic without increasing or bringing other comorbidities; however, we did not expect to improve functionality since the patient had completely lost it for years and she agreed with this risk prior to the intervention.

Setting realistic objectives and maintaining truthful communication with patients is essential since it is difficult for the results to utopically encompass all sensory and motor needs without adverse effects. That is why carrying out an exhaustive evaluation before deciding on an IBIP is necessary - not to offer relief from all discomfort but a significant improvement in the quality of life of patients who are candidates for this type of treatment.

## Conclusions

The implementation of IBIP has proven to be an effective and safe treatment to achieve pain relief goals associated with painful spastic hemiplegia secondary to stroke. This case illustrated the possibility of considering this option despite the chronicity of the deficit over the past 15 years. However, this treatment offers limited improvement in motor skills and is not exempt from adverse effects, so establishing realistic goals together with the patient can turn the IBIP into a treatment aimed at improving specific conditions of spasticity associated with stroke and its functional complications.
